# Lifestyle intervention and support preferences to maximize health outcomes in adolescent bariatric surgery patients

**DOI:** 10.1017/cts.2024.553

**Published:** 2024-09-19

**Authors:** Marlyn A. Allicock, Rashon King, Jackson Francis, M. Sunil Mathew, Dhatri Polavarapu, Alicia Wheelington, Maral Misserian, Bethany R. Cartwright, Adejumoke Adewunmi, Aparajita Chandrasekhar, Faisal G. Qureshi, Sarah E. Barlow, Sarah E. Messiah

**Affiliations:** 1 The University of Texas Health Science Center at Houston School of Public Health, Dallas, TX, USA; 2 Center for Pediatric Population Health, UTHealth School of Public Health, Dallas, TX, USA; 3 Children’s Health System of Texas, Dallas, TX, USA; 4 School of Health Professions, University of Texas Southwestern Medical Center, Dallas, TX, USA; 5 Department of Pediatrics, University of Texas Southwestern Medical Center, Dallas, TX, USA; 6 Touchstone Diabetes Center, University of Texas Southwestern Medical Center, Dallas, TX, USA; 7 Department of Surgery, University of Texas Southwestern Medical Center, Dallas, TX, USA; 8 Department of Pediatrics, UTHealth McGovern Medical School, Houston, TX, USA

**Keywords:** Adolescent, metabolic and bariatric surgery, weight loss surgery, lifestyle intervention, weight loss

## Abstract

**Introduction:**

Metabolic and bariatric surgery (MBS) is safe and efficacious for adolescents with severe obesity. Pairing MBS with behavioral lifestyle interventions may be effective for optimizing treatment outcomes. However, no standardized program exists. Adolescent perspectives are critical to understanding how to design interventions to enhance engagement, sustain motivation, and meet informational needs for pre- and post-MBS self-management behaviors. The aim of this study was to develop an MBS lifestyle support intervention built on evidence-based content with input from adolescents and their families.

**Methods:**

A mixed-methods design identified adolescent preferences for MBS lifestyle support. Data were collected from a racially and ethnically diverse sample of adolescents (*N* = 17, 76% females, 24% males 41.2% non-Hispanic Black, 41.2% Hispanic/Latino, 11.8% non-Hispanic White, 5.8% Other) and their mothers (*N* = 13, 38.4% Hispanic) recruited from an MBS clinic. Quantitative surveys and qualitative interviews assessed preferred types of pre-post MBS content, modality, frequency, and delivery platforms to inform the design of the intervention. Mixed methods data were triangulated to provide a comprehensive understanding of adolescent/parent preferences.

**Results:**

Adolescents prioritized eating well, managing stress, and maintaining motivation as desired support strategies. Parents identified parental support groups and nutrition guidance as priorities. Peer support and social media platforms were identified as key approaches for boosting motivation and engagement.

**Conclusions:**

The patient voice is an important first step in understanding how, and whether behavioral lifestyle programs combined with MBS for weight management can be optimized. Adolescent preferences may enhance program fit and identify health behavior supports needed to sustain behavior change.

## Introduction

The prevalence of severe obesity among United States adolescents has almost doubled from 5.2% to 9.2% over the past decade [1]. Severe obesity during adolescence is associated with cardiometabolic and mental health comorbidities, liver and kidney disease, osteoarthritis, and lower sleep quality, resulting in lower quality of life [2,3]. Metabolic and bariatric surgery (MBS) is safe and efficacious in treating adolescents with severe obesity [4,5]. A recent American Academy of Pediatrics (AAP) policy statement [6] highlighted the need for better access to MBS for adolescents with severe obesity when medically indicated. Additionally, subsequent AAP clinical practice guidelines have supported this policy [7]. Despite its effectiveness in promoting weight loss, MBS alone does not adequately prevent weight regain following surgery and must be supported by lifestyle change. However, studies show that adolescents have suboptimal postoperative adherence to behavioral and lifestyle recommendations [8]. To date, there is no standardized healthy lifestyle behavior intervention for adolescent MBS patients and their families designed to address adolescent pre- and post-operative needs.

The Diabetes Prevention Program Group Lifestyle Balance (DPP/GLB) [9] was created to address pre-diabetes/type 2 diabetes (a common comorbidity among MBS patients) among adults and is built on decades of NIH-supported research. Central to DPP/GLB is the Centers for Disease Control and Prevention-recognized lifestyle change program, focused on healthy eating and physical activity, which has been shown to lower the risk of developing type 2 diabetes by 58% among adults [10]. Yet, the DPP/GLB program has never been coupled with MBS to support sustained weight loss. The DPP/GLB may be a promising evidence-based intervention to adapt for weight management among adolescents who undergo MBS. However, there is a knowledge gap regarding adolescent educational information and needs for support pre- and post-MBS surgery. Increasing our understanding of adolescents’ informational and support preferences may contribute to improvements in clinical care post-MBS. Patient perspectives [11] can provide valuable information to improve healthcare, challenge ingrained practices, and integrate expertise and real-world experiences that exist outside of the medical setting. Thus, we aimed to obtain the perspectives of adolescents considering MBS, and their parents, and to use the information to inform the adaptation of DPP/GLB content information, design, and modality of a lifestyle intervention for adolescents pre- and post-MBS.

## Methods

### Study design

Using a mixed-methods study design that included a survey and semi-structured qualitative interviews, we examined adolescent preferences regarding content information, modality delivery, and design to inform the adaptation of an MBS behavioral intervention for adolescents. We surveyed parents about their needs and providing support to adolescents. Conceptual frameworks that guide intervention development for adolescents with severe obesity and MBS are lacking. Others [12] have recommended using ecological approaches to understand and influence weight management strategies. As such, the socioecological model (SEM) [13,14] that describes behavioral level influences (intrapersonal, interpersonal, group/ community, societal/environmental) for weight management guided our approach. Additionally, Social Cognitive Theory (SCT) [15], which explains human behavior as a dynamic, reciprocal model in which personal factors, individual influences, and behaviors continually interact, was also applied. SCT was particularly appropriate as it focuses on learning skills for weight management rather than solely on knowledge acquisition. Thus, our questions asked about barriers/facilitators to SEM/ SCT intrapersonal constructs (e.g., body image), interpersonal factors (e.g., peer influence on the decision to have MBS/ weight loss after MBS), and group/community constructs (e.g., MBS support system(s), discrimination experiences, food/ activity environments to support MBS), and macro/policy level (media influences, environmental support for post-MBS lifestyle).

### Participants

Adolescents and parents at the Center for Obesity and its Consequences in Health (COACH) and Weight Management clinics at Children’s Health were recruited. Clinic staff provided patient lists in EPIC and study staff contacted parents to confirm eligibility and willingness to participate. If parents were interested and verbally confirmed that the adolescent met inclusion criteria, they were sent the quantitative survey via Qualtrics. This survey contained an e-consent and assent. Parents consented first, and adolescents assented after their parents gave permission. Adolescents were eligible if they met the National Institute of Health criteria for MBS [16], could communicate in English, and were ages 13–18 years.

### Procedures

Interviews and surveys were conducted in English from May 2022 to June 2023. Data collection was completed in parallel. All study procedures were approved by the institutional review boards at The University of Texas Health Science Center at Houston, The University of Texas Southwestern Medical Center, and Children’s Medical Center Dallas prior to parental consent (and participant assent, as applicable). Consent forms were in English and Spanish.

### Quantitative survey description

Adolescents completed a brief survey on Qualtrics that collected demographic information (age, gender, race/ethnicity, education level, living with parent or independently) and explored their preferences for a support program (i.e., modality, frequency, timing, and delivery platforms) offered pre- and post-MBS. The child survey specifically queried about MBS stage; food and exercise; websites, platforms, and apps used for weight management support; type and frequency of support before and after MBS; and DPP/GLB content preferences. At the end of the survey, a question asked whether participants were interested in a follow-up interview or focus group to learn more about their weight management and support needs. Interested participants, all of whom preferred interviews, were contacted by the study staff. Information regarding adolescent co-morbidities was obtained from the electronic health record. Participants did not receive compensation for completing the survey but did receive a $25 Amazon gift card if they completed an interview. Parent surveys were collected at the same time as adolescent surveys. Parent demographic information was collected (age, gender, race/ethnicity, insurance status, income). The parent survey explored parental preferences for a support program and ways they would support their child.

### Qualitative interview description

**Figure 1.**
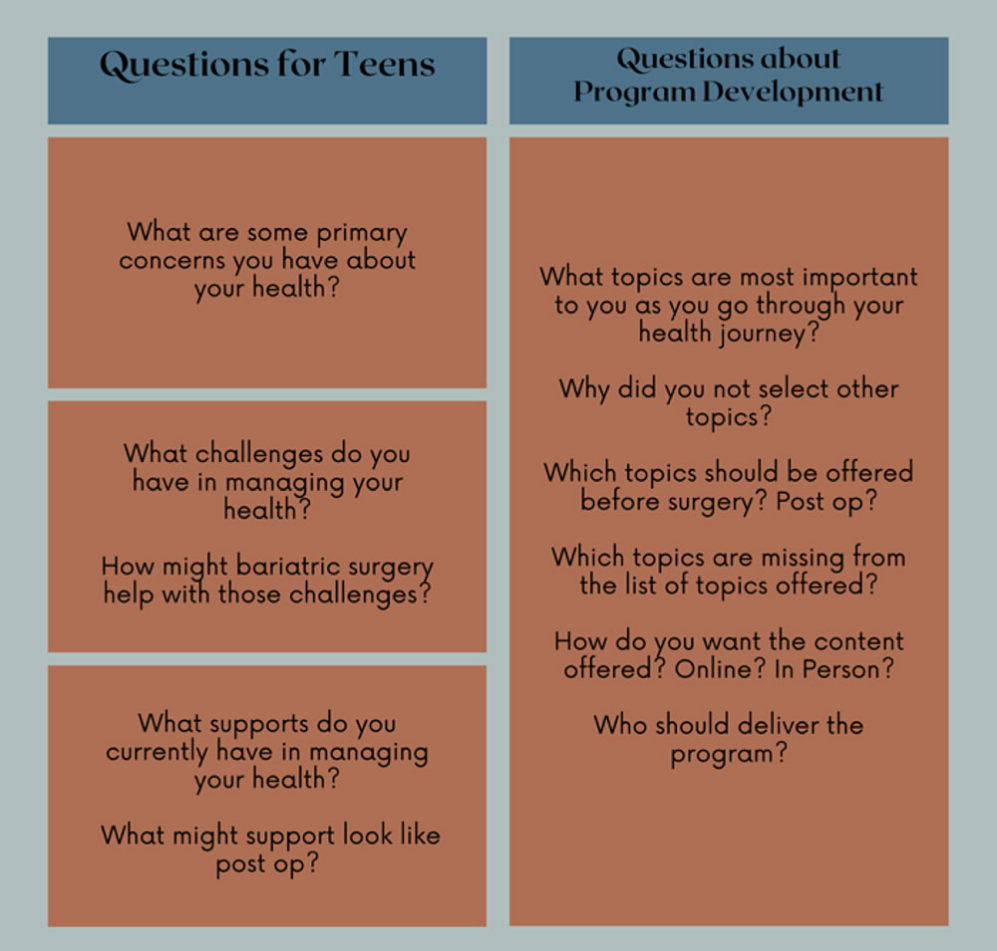
Qualitative interview guide.

The semi-structured interview was guided by the SEM/SCT constructs and queried participants about their pre-MBS health journey and explored their decisions and desires regarding weight loss and support (Figure 1). Participants also discussed their preferred topics from the DPP/GLB modules and content delivery. Interviews were conducted by trained study staff without a clinical relationship via ZOOM/Microsoft Teams and were digitally recorded. Interviews lasted 15–25 minutes.

### Analysis

Descriptive summary statistics, including frequencies, means, and standard deviations, were calculated for all quantitative survey outcomes. Each participant selected all topics that were of interest from the list of thirteen DPP/GLB topics that the clinic team deemed appropriate for this age group. They then ranked their selections which ranged from 1st to 8th. Rankings were converted into scores, with the top choice receiving a value of “8,” the second choice a “7,” and so forth until all topics were scored. The scores for each participant were subsequently totaled to identify the modules with the highest overall scores. Survey data were analyzed using SAS (SAS Institute, Cary, NC) version 9.4.

Qualitative interviews were professionally transcribed, and data analyzed using NVivo 12.0 (QSR International, AUS). Interview coding was conducted by three team members (RK, JF, AA) independently. Each member started by individually coding each interview to define key concepts and develop a preliminary code structure based on the first three transcripts and each transcript was reviewed by a secondary reviewer using an iterative process. Following interview coding, thematic analysis was conducted by two team members (RK, JF). Using the constant comparative approach, we applied our code structure to all transcripts and revised codes as new concepts emerged [17,18]. The resulting themes were regarded as “saturated” by the data analysts [18]. Any discrepancies in coding were decided through research team consensus [18]. Both survey and interview data were collected in parallel, analyzed separately, then merged to augment the interpretation of the findings [19] to support the adaption of DPP/GLB content to better align with adolescents. For example, we reconciled data across methods (qualitative and quantitative) [20] by examining the ranking of DPP topics on the survey and comparing with the topics identified in the qualitative interviews as most important and not at all important to understand in greater detail why topics may be ranked as such. All data were used to support the development and implementation of a support program for adolescents pre-and post MBS. We report on the patient and parent preferences here and not about the developed program.

## Results

**Figure 2.**
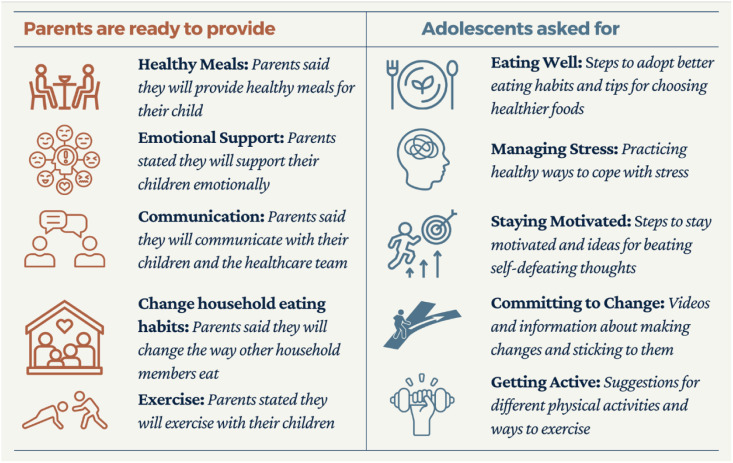
Parental support and adolescent needs.

Seventeen participants (mean age 15.53 years SD 1.33 years, range 13–17 years, 76.5% female, 41.2 % Black, 41.2% Hispanic, 11.8% white, Other 5.8% ) completed the survey (Table 1). The majority (70.6%) of participants were pre-surgery, 11.8% were post-surgery, and 17.6% were undecided about completing MBS. A total of 15 (88.2%) participated in semi-structured qualitative interviews. A total of 13 mothers completed the parent survey (61.6% non-Hispanic, 38.4% Hispanic) (Table 2). No parent interviews were collected. Figure 2 summarizes adolescent and parent findings.

**Table 1.** Demographics of adolescent participants

Demographics, *N* (%)	Survey participant (*N* = 17)	Interview Participants (*N* = 14)
**Age (13–17), mean (sd)**	15.53 (1.33)	15.35 (1.44)
**Sex, *n* (%)**		
Male	4 (23.5)	4
Female	13 (76.5)	10
**Race/Ethnicity, *n* (%)**		
Non-Hispanic Black	7 (41.2)	6
Non-Hispanic White	2 (11.8)	3
Hispanic	7 (41.2)	5
Other	1 (5.8)	0
**Education Level**, ***n* (%)**		
In middle school	2 (12.0)	3 (21.4)
In high school	14 (82.0)	11(78.6)
Other	1 (6.0)	
**BMI, mean (range)** BMI greater than or equal to 95^th^ percentile*	98.9 (97.7–99.74)	99.5 (99.21–99.74)
**MBS status, *n* (%)**		
In process of completing requirements for surgery	12 (70.6)	11 (73.3)
Completed Surgery	2 (11.8)	1 (13.3)
Undecided	3 (17.6)	2 (13.3)
**Comorbidities, *n***		
Hypertension	1	3
Diabetes	3	0
Sleep apnea	9	11
Gastroesophageal reflux	3	2
Polycystic Ovarian Syndrome	1	0
Dyslipidemia	0	0
Non-alcoholic fatty liver disease	0	0
**Insurance Status**		
Public	10 (58.8)	8 (57.2)
Private	3 (17.6)	3 (21.4)
Unknown	4 (23.6)	3 (21.4)

*BMI = body mass index; MBS = metabolic and bariatric surgery*.

**Table 2.** Demographics of parents

Demographics, *N* (%)	Survey participant (*N* = 13)
**Age (35–72), mean, (sd)**	44.5 (9.89)
**Sex, *n* (%)**	
Female	13 (100)
**Race, *n* (%)**	
Black	6 (46.2)
White	6 (46.2)
Other	1 (7.6)
**Ethnicity, *n* (%)**	
Non-Hispanic	5 (38.4)
Hispanic	8 (61.6)
**Insurance Status**	
Public	10 (69.2)
Private	3 (30.8)
None	0
**Household Income**	
Less than 10,000	1
10–15,000	2
15–20,000	0
20,000–25,000	0
35–50,000	2
50–75,000	1
75–100,000	0

### Preferences for intervention delivery, format, and modality

#### Adolescent survey findings

Adolescents chose in-person support groups, followed by live web-based support options including Zoom or Facetime, and mobile apps as their preferred method of intervention delivery (Table 3). Almost half of participants (47%) indicated support should be offered pre- and post-MBS in 30–45-minute sessions. Adolescents (59%) reported that they would use a health management app to aid in their health journey pre- and post-MBS, daily or most days of the week, 24% several times a week, and 6% weekly or less frequent usage. Most preferred educational receiving content via smartphones or social media.

**Table 3.** Adolescent preferences for pre-and post metabolic and bariatric surgery (MBS) weight-management support

	Survey Participants (*N* = 17)
**Support Tools used currently for Weight Loss**	
Support group (in-person or web-based)	0
Social media group (Facebook, etc.)	0
Mobile/tablet app	**5**
Other	0
No tools used	12
Support Tools would like for weight loss pre-MBS*	
Support group (in-person)	**7**
Live web-based support group (web conference, FaceTime, chatroom)	5
Social media group (Facebook, etc.)	4
Mobile/tablet app	5
Other	0
None	1
Support Tools would like for weight loss post-bariatric surgery*	
Support group (in-person)	**6**
Live web-based support group (web conference, FaceTime, chatroom)	5
Social media group (Facebook, etc.)	3
Mobile/tablet app	5
Other	0
None	1
**Timing of Support**	
Before surgery only	1
After surgery only	2
Before and after surgery	**8**
No response given	6
**Preferred Length of Support Sessions**	
15 minutes	2
20 minutes	1
30 minutes	2
45 minutes	**3**
No response given	**9**
**Preferred Frequency of Use for social media, apps, other eHealth tools**	
Daily or most days	**10**
A few times/week	4
Once a week or less	1
No response given	2
Preferred Platforms for healthy lifestyle support pre/post-surgery*	
Smartphone	**13**
Tablet	1
Website	4
TV	1
Gaming console	3
Social media	6

*Counts provided; participants were given the option to select more than one choice.

#### Adolescent qualitative findings

Interview responses on delivery preferences varied. Some emphasized the convenience of online content (i.e., without live in-person/virtual delivery), while others expressed concerns about reduced accountability and engagement in online formats. One *explained, “ I tend to get unfocused if you’re not actually right in front of me” (15-year-old, Non-Hispanic White, female).* When asked about effective mediums for content delivery, overwhelmingly, adolescents preferred frequented social media platforms such as TikTok and Instagram. Peer support was cited as important for in-person and online content delivery. For example, adolescents emphasized that seeing other adolescents on a similar journey would be motivating. A few felt it would be helpful to see someone their age who completed MBS deliver the program content. However, most preferred that content be administered by medical professionals such as their pre-op psychologist or surgeon.

*“I think the different people at different times would be good because I know it would help me, and it’ll probably help other people to have somebody around the same age as me who went through this experience at this age, had these problems, and to see what it looks like with them having gone through it now. And also having the doctor to show why this is important and why you’re doing it.” (15-year-old, Non-Hispanic White female)*

### DPP/GLB topic survey ranking and interest

#### Adolescent survey findings

Table 4 displays participants’ rankings for their preferred topics from the list of thirteen DPP/GLP modules. “Eat Well” was the top choice, signifying the strong inclination of participants toward information and guidance on healthier eating habits and food choices. “Managing Stress” and “Stay Motivated” closely followed. Other highly rated modules included “Commit to Change,” and “Get Active.” In the mid-range, topics include “Get Back on Track,” “Take a Fitness Break,” “Eat Well Away from Home,” “Food Tracking,” and “Activity Tracking.” Conversely, modules like “Get Support,” “When Weight Loss Stalls,” and “Check-In and Keep Going” received the lowest scores.

**Table 4.** Adolescent preferred diabetes prevention program modules ranking list

Module Topic	Score
**Eat Well** *Contains steps to adopt better eating habits and tips for choosing healthier foods*	**66**
**Managing Stress** *Practice healthy ways to cope with stress*	52
**Stay Motivated** *Offers steps to stay motivated and ideas for beating self-defeating thoughts*	52
**Commit to Change** *Contains testimonial videos and information about the program’s efficacy and is tailored to your age group*	51
**Get Active** *Get suggestions for different physical activities based on your preferences*	50
**Activity Tracking** *Provides concrete steps that encourage you to track physical activity and options to meet weekly activity goals*	37
**Food Tracking** *Information about developing a food log and tracking the foods you eat*	36
**Eat Well Away from Home** *Helps with selecting healthy food choices away from home*	30
**Take a Fitness Break** *Practice quick exercises in movement and how to work these activities into your daily life*	24
**Get Back on Track** *Provides solutions, a step-by-step plan, and encouragement on your weight loss journey*	20
**Check-In and Keep Going** *Provides habits of others who have successfully reached their goals*	13
**When Weight Loss Stalls** *Helps you stay motivated in your weight loss journey*	12
**Get Support** *Provides suggestions on how to find social support for positive changes*	**0**

#### Adolescent interview findings

Participant responses regarding content mirrored survey responses. “Physical activity” emerged as an important theme, underscoring its pivotal role in both weight loss and overall fitness. *“I think that if people get the surgery, they should stay active….like especially me, if I’m going to get the surgery then I’m going to need to stay active…”* (14-year-old Non-Hispanic White male). There was an emphasis on “eating well,” a major challenge adolescents identified in maintaining a healthy diet pre- and post-op. *“I would just like to know things to make or to know to not eat like greasy food or just know to make better things instead of making them the bad way.”* (16-year-old, Hispanic White female). The theme of “eating outside the home” reflected the real-world challenges of maintaining a well-balanced diet while in social settings.

“*Well, sometimes you go to the store, and you see all the good foods, like all the junk foods and all the cupcakes and candy. And it’s just kind of hard to get away from it… And then, find some things that are lower calorie that are… say, low-calorie desserts, so I do not have to only eat jello and sugar-free jello all that time if I want something sweet.”* (17-year-old, Hispanic/Latina White female).

We asked about topics not selected. While most noted that those topics would be generally helpful, two adolescents explained that the topics not selected did not stand out because of their prior knowledge and current support for these topics.

*“…I didn’t pick the others because they weren’t really relevant to me because I already understood how to cope and I know what to do. And I know, if I do have a problem, I can just go to someone or ask questions… eating-wise, I kind of have understanding about. I have an uncle and he just knows so much about that, so I can always just go to him. And also, about being fit or moving around, I can just ask him.*” (15-year-old, Hispanic/Latina female).

### Family support for weight management support interventions

Adolescents provided information through interviews and parents through surveys about the role of family support.

#### Adolescent interviews

Adolescents expressed a collective desire that support programs acknowledge family influence on their behaviors and offer strategies to support families and adolescents to stay on track with behavioral changes. Particularly, attention to cultural family habits (e.g., eating patterns) should be considered. Adolescents advocated for programs to address challenges of unsupportive family members and eating healthy away from home.

“*… if you live with people who do not really care about losing weight, they buy chips, candy, or say your mom’s Southern, your grandma’s Southern like mine is, and really for probably my whole life every other night, she made fried chicken. So, maybe trying to help people’s families, especially teenagers. Getting the family involved and trying to make a whole switch or trying to keep those kind of items out the house.” (*17-year-old, Hispanic/Latina female).

#### Parent survey responses

There were 13 survey responses of which all were the mothers of adolescents (Table 5). 85% of parents knew someone who had MBS. 62% of parents reported that, if available, they would participate in a support group solely for parents of children who had MBS. 69% of parents had health insurance coverage for the bariatric procedure. Parents reported being willing to exercise with their child (85%), provide emotional support (100%), advocate for child (77%), and change drinks (85%) and meals (69%) served at home. Parents reported that eating outside of the home (77%) and not knowing healthy recipes to prepare (77%) were challenges for supporting their child’s weight loss goals.

**Table 5.** Parent survey responses

Survey Item	Survey participant (*N* = 13)
Knowledge of metabolic and bariatric surgery (MBS)	
Yes, prior knowledge	11 (85.0)
No, no prior knowledge	2 (15.0)
Bariatric Surgery Concerns*	
Safety	7
Recovery	9
Side effects	6
Future complications	6
Medications taken post op	3
Cost	4
Food options post op	7
No concerns/issues	1
Cause of weight of child as child’s responsibility	
Yes	4
No	8
Maybe	1
Parent Support for Child*	
Exercise with child	11
Be emotionally supportive	13
Advocate for my child’s needs	10
Communicate with healthcare team	13
Change foods/drinks in household	11
Change meals eaten by other household members	9
Provide healthy meals/options	11
Support Group only for Parents	
Yes, I would participate	5
Maybe, I would participate	8
No, I would not participate	0
Parental Support System Preferences*	
In Person Support Group	4
Web based support group	6
Social Media Group (Facebook, etc)	6
Mobile tablet/app	7
None	1
Current Use of Support tools to Aid in Child’s Weight Loss	
Yes	4
No	9
Topics of Interest to Know About to Support Weight Loss*	
Information on Healthy Options in restaurants	6
How to read food labels	2
Places to exercise	6
Healthy recipes	10
Strategies for healthy portions	6
Changes in meals (timing, content, portion) that result from surgery	9
How to handle eating outside the home after surgery	10
Potential nutrition problems after surgery	9

*Counts provided; participants were given the option to select more than one choice.

## Discussion

There are currently no standardized behavioral supports available for adolescents who are undergoing MBS to sustain weight loss and adopt healthy behaviors. Other studies with adolescents post-MBS have identified the need for support post-operatively [21–23]. In adult populations, particularly the first year post-surgery is a critical time when patients need support [23]. Given the dearth of literature focused on behavioral interventions to support adolescents pre- and post-MBS, we sought to explore their needs and preferences. Currently, the Diabetes Prevention Program Group Lifestyle Balance (DPP/GLB) is offered in person for adults. The DPP/GLB could provide a model of behavior support, given its success among adults with pre-diabetes, but it has not been studied in adolescents, and adaptation would benefit from qualitative exploration of adolescent perspectives. Adolescents voiced critical support needs and identified aspects of the DPP/GLB program that could be adapted to support behavioral management for those seeking MBS. We found that adolescents and their parents endorsed the need for support before and after surgery, although adolescents preferred having in-person encounters prior to MBS versus virtual/technology-supported interactions post-MBS. Adolescents also expressed general consensus that a medical professional deliver these programs, with examples of peers highlighted. Parents also indicated they would benefit from having a parent support group. Parents wanted to know how best to navigate eating out and identify healthy recipes. Given that the family environment impacts adolescent obesity through food availability, meal structuring/planning, cultural preferences, and engagement in physical activity [24,25], parental supports are also needed.

Adolescents’ top-rated DPP/GLB modules included “Eat Well,” “Managing Stress,” and “Stay Motivated” were identified by survey and interview participants as essential content for weight management support. These preferences reflect similar findings of adult patients managing obesity who reported stress management, goal setting, and staying motivated were topics they wanted for weight loss [23]. Making adolescent preferred modules the program’s focal points ensures alignment with the preferences and requirements of adolescents contemplating MBS, which could foster engagement and compliance.

Lower-ranked DPP/GLB topics such as “Food Tracking,” and “Activity Tracking,” still offer value. Adolescents noted in the interviews that these topics were generally helpful, but because they had prior knowledge and current support, they did not need additional programmatic support. Adolescents preparing for MBS have likely had opportunities to engage with content relevant to dieting and physical activity. Thus, it may be reasonable to suggest that topics related to activity tracking and food tracking, for example, were not as prominent, given that they may already have had experience with those behaviors and less focus on managing stress or ways to stay motivated. Given that our participants cited having family and friends’ support, getting social support was the least frequently selected DPP/GLB topic. However, they suggested they needed guidance to navigate situations when family members were not supportive of their healthy behaviors.

Social media platforms were identified as a viable option for program delivery. This is not surprising given that a Pew 2022 survey [26] reported that YouTube, TikTok, and Instagram were the most popular platforms used by 95% of adolescents. During the interviews, adolescents discussed the frequency and ease of social media platforms for garnering information. This suggests that future support programs designed for MBS patients may be best offered on these platforms. These findings reflect similar results from a study exploring social media preferences for healthy weight among non-Hispanic Black and Hispanic/Latino adolescents that identified TikTok as a preferred platform for learning new information about healthy weight and TikTok and Instagram for connecting and offering support for healthy weight management [27]. Although adolescents in our study did not share any apprehension or concerns regarding privacy or misinformation using social media platforms, these are issues that programs should consider in any program design.

Outside of social media platforms, apps may be useful in administering content given how useful adolescents find apps when managing their health. M FC;ssener and colleagues (2022) sought input from adolescents and healthcare providers about using an app to offer preoperative and postoperative MBS support through a mHealth intervention focused on improving long-term healthy behaviors and health outcomes [28]. The consensus was that a mHealth tool such as an app should complement physical appointments, offer individually tailored support, track appointments, and include brief and easy-to-understand support information [28]. Other features to enhance engagement in health promotion to support health goals among adolescents have included gamification studies [29–31]. A recent systematic review of 42 studies [32] noted that mHealth for pediatric weight management is still in its infancy, with most studies focused on the feasibility, acceptability, or usability of mHealth interventions. This further highlights the need to capture patient preferences to inform this emerging evidence base regarding support for adolescents undergoing MBS.

The message source for weight management information was identified as critical for adolescent engagement. Regardless of whether support was provided pre- or postoperatively, participants endorsed the need for health-related content to be provided by a health professional. Trustworthy healthcare information for patients is viewed as provided by healthcare professionals [33]. However, our participants, as in other pediatric weight management studies [34,35], also favored seeing/hearing from peers who had completed MBS to learn about their experiences and gain motivation. Others have suggested that future research identify how best to leverage the experiences of adolescents who have had MBS to educate and support other patients considering MBS [36]. Programs should include peers who can model behaviors, share strategies for overcoming challenges, and success stories.

### Study limitations

Our study had some limitations. Our sample, while diverse, was not evenly distributed by gender. Only 24% of participants were males, and our findings may not account for potential sex differences in desired preferences. However, this representation is consistent with other adult and adolescent MBS studies. It is important to point out that there is variation in adolescent obesity rates by region, state, and socioeconomic status in addition to the noted variation by age, gender, race, and ethnicity. As such, adolescents are not a homogenous group, and additional considerations may be appropriate when designing interventions for this group. Although surgery status could contribute to differences in adolescents’ and parents’ opinions about what could potentially be helpful to include in a program, we noted no differences in responses from participants who were pre-operative vs. the two participants who had previously had surgery. Additionally, most interviews were completed while adolescents were with or near a parent, which may have impacted how adolescents responded to questions and reflected a social desirability bias.

## Conclusions

The adolescent patient voice will likely improve the utility and appeal of pre-and post MBS weight management support content and strategy development. Adolescent-endorsed content focused on healthy eating, managing stress, and maintaining motivation to support self-management behaviors and preferred programs delivered via their preferred social platforms. Adolescents expressed a preference for peer models who had completed MBS to assist them in navigating their weight management journey. Parents are essential in supporting their adolescent’s health, and they, too, seek support for caring for their children. Thus, MBS support programs should consider incorporating parental strategies. This study contributes to the growing body of research focused on weight management among this patient population, identifying factors relevant for enhancing engagement and adherence to weight management programs. Adolescent insights provide practical implications for what content is relevant, who should provide education, and what formats they are likely to engage in that can optimize support offered by their clinical teams to maximize post-MBS weight loss and health outcomes. Given the paucity of research focused on adolescent perspectives on behavioral support interventions, further research regarding educational content, modality, and frequency of support would improve obesity treatment interventions and outcomes.
